# “Follow the Leader”: A Centrality Guided Clustering and Its Application to Social Network Analysis

**DOI:** 10.1155/2013/368568

**Published:** 2013-10-24

**Authors:** Qin Wu, Xingqin Qi, Eddie Fuller, Cun-Quan Zhang

**Affiliations:** ^1^Department of Computer Science, Jiangnan University, Wuxi, Jiangsu 214122, China; ^2^Department of Mathematics, West Virginia University, Morgantown, WV 26505, USA; ^3^School of Mathematics and Statistics, Shandong University at Weihai, Weihai 264209, China

## Abstract

Within graph theory and network analysis, centrality of a vertex measures the relative importance of a vertex within a graph. The centrality plays key role in network analysis and has been widely studied using different methods. Inspired by the idea of vertex centrality, a novel centrality guided clustering (CGC) is proposed in this paper. Different from traditional clustering methods which usually choose the initial center of a cluster randomly, the CGC clustering algorithm starts from a “LEADER”—a vertex with the highest centrality score—and a new “member” is added into the same cluster as the “LEADER” when some criterion is satisfied. The CGC algorithm also supports overlapping membership. Experiments on three benchmark social network data sets are presented and the results indicate that the proposed CGC algorithm works well in social network clustering.

## 1. Introduction

Clustering is a process of partitioning a set of data into meaningful subsets so that all data in the same group are similar and the data in different groups are dissimilar in some sense. It is a method of data exploration and a way of looking for patterns or structure in the data that are of interest. Clustering has wide applications in social science, biology, chemistry, and information sciences. A general review of cluster analysis can be found in many references such as [1–4].

The commonly used clustering methods are partitional clustering and hierarchical clustering. Partitional algorithms typically determine all clusters at once. *K*-means [5] clustering algorithm is a typical partitional clustering. Given the number of clusters (say *k*), the procedure of *K*-means clustering is as follows. (i) Randomly generate *k* points as cluster centers and assign each point to the nearest cluster center. (ii) Recompute the new cluster centers. (iii) Repeat the two previous steps until some convergence criterion is met. The main advantages of the *K*-means algorithm are its simplicity and speed which allows it to run on large datasets. However, it does not yield the same result with each run, since the resulting clusters depend on the initial random assignments. And the number of clusters has to be predefined.

The hierarchical clustering is either agglomerative or divisive. Agglomerative algorithms begin with each element as a separate cluster and two clusters separated by the shortest distance are merged successively. Most hierarchical clustering algorithms are agglomerative, such as SLINK [6] for sing linkage and CLINK [7] for complete linkage. Divisive starts with one big cluster and splits are performed recursively as one moves down the hierarchy. The hierarchical clustering builds a hierarchy tree of clusters, which is called dendrogram. The way in which elements are clustered is clearly shown in the dendrogram.

In recent years, social network analysis has gained much attention. Social network analysis is the study of social relations in terms of networks. A social network is usually modeled as a directed graph or undirected graph. The set of nodes in the graph represent individual members. The set of edges in the graph represent relationship between the individuals, such as friendship, coauthorship, and so forth. A fundamental problem related to social networks is the discovery of clusters or communities. Porter et al. [8] summarized different clustering methods for social network clustering. Wu and Huberman [9] proposed to find communities based on notions of voltage drops across networks. Girvan and Newman [10] proposed to discover community structure based on edge betweenness. Newman [11] proposed to find community structure based on the eigenvectors of matrices. Clauset et al. [12] proposed a modularity-based method for finding community structure in very large networks.

In this work, a novel hierarchical clustering algorithm is proposed for social network clustering. Traditional clustering methods, such as *K*-means, usually choose clustering centers randomly, and the hierarchical clustering algorithms usually start from two elements with shortest distance. Different from these methods, this work chooses the vertex with highest centrality score as the starting point. If one does some analysis on social network datasets, one may notice that in each community, there is usually some member (or leader) who plays a key role in that community. In fact, centrality is an important concept [13] within social network analysis. High centrality scores identify members with the greatest structural importance in a network and these members are expected to play key roles in the network. Based on this observation, this work proposes to start clustering from the member with highest centrality score. That is, a group is formed starting from its “leader,” and a new “member” is added into an existing group based on its total relation with the group. The main procedure is as follows. Choose the vertex with the highest centrality score which is not included in any existing group yet and call this vertex a “LEADER.” A new group is created with this “LEADER.” Repeatedly add one vertex to an existing group if the following criterion is satisfied: the density of the newly extended group is above a given threshold.

The paper is organized as follows. Different centrality measurements are discussed in Section 2. The proposed clustering algorithm is described in Section 3. In Section 4, test results of the new algorithm on some social network bench-mark datasets are compared with ground truth and some traditional methods. Conclusions are made in Section 5.

## 2. Measures of Centrality

Centrality is one of the most widely studied concepts in social network analysis. Within graph theory and network analysis, centrality of a vertex measures the relative importance of a vertex within the graph. For example, how important a person is within a social network or how well used a road is within an urban network. During past years, various measures of the centrality of a vertex have been proposed. Centrality measurement, such as degree centrality, betweenness, and eigenvector centrality, are among the most popular ones.

Degree centrality is the simplest centrality measurement. Given a graph *G*, denote the set of vertices of *G* as *V*(*G*), and then the degree centrality for any *v* ∈ *V*(*G*) is defined as

(1) $$\begin{array}{c} C_{D}\left(v\right)=\frac{d\left(v\right)}{\left|V\left(G\right)\right|-1}, \end{array}$$

where *d*(*v*) is the degree of *v* and |*V*(*G*)| is the number of vertices in *G*.

Degree centrality considers only the local topology of the network. It can be interpreted as a measure of immediate influence, as opposed to global effect in the network [14].

The betweenness centrality for any *v* ∈ *V*(*G*) is defined as

(2) $$\begin{array}{c} C_{B}\left(v\right)=\frac{2}{\left(\left|V\left(G\right)\right|-1\right)\left(\left|V\left(G\right)\right|-2\right)}\;\underset{s\neq v\neq t}{\sum }\;\frac{\sigma_{st}\left(v\right)}{\sigma_{st}}, \end{array}$$

where *s*, *v*, *t* ∈ *V*(*G*), *σ*_*st*_ is the number of shortest paths from *s* to *t*, and *σ*_*st*_(*v*) is the number of shortest paths from *s* to *t* that pass through the vertex *v*.

Betweenness centrality is one of the most popular centrality measures which consider the global structure of the network. It characterizes how influential a vertex is in communicating between vertex pairs [15].

The eigenvector centrality score of the *i*th vertex in the network is defined as the *i*th component of the eigenvector corresponding to the greatest eigenvalue of the following characteristic equation:

(3) $$\begin{array}{c} Ax=\lambda x, \end{array}$$

where *A* is the adjacency matrix of the network, *λ* is the largest eigenvalue of *A*, and *x* is the corresponding eigenvector. It simulates a mechanism in which each vertex affects all of its neighbors simultaneously [16].

Eigenvector centrality is a sort of extended degree centrality which is proportional to the sum of the centralities of the vertex's neighbors. A vertex has large value of eigenvector centrality score either if it is connected to many other vertices or if it is connected to others that themselves have high eigenvector centrality [17].

Due to the fact that different centrality measures are based on different aspect of a network, the final centrality scores and ranking of the nodes in the network may be different. The difference will be discussed in Section 4.

## 3. Centrality Guided Clustering

In this section, some notation and terminology are introduced and the centrality guided clustering (CGC) algorithm is presented.

Given an input dataset, the dataset is modeled as a weighted graph *G* = (*V*, *E*, *w*). *V* is the vertex set. Each vertex in *V* represents an element in the dataset. |*V*(*G*)| represents the number of vertices in *G* (or elements in the dataset). *E* is the edge set. Each edge represents a relationship between a pair of elements. *w* is the edge weight function. *w*(*u*, *v*) and *w*(*e*) denote the weight of the edge *e* between two vertices *u* and *v*. If there is no edge between two vertices *u* and *v*, then *w*(*u*, *v*) = 0. If the graph is an unweighted graph, then

(4) $$\begin{array}{c} w\left(uv\right)=\{\begin{array}{cc} 1, & \text{if}\;uv\in E\left(G\right),\\ 0, & \text{if}\;uv\notin E\left(G\right). \end{array} \end{array}$$

Let *C* be a subgraph of *G*, the density of the subgraph *C* is defined as

(5) $$\begin{array}{c} \text{density}\left(C\right)=\frac{2\sum_{e\in E(C)}w\left(e\right)\;}{\left|V\left(C\right)\right|\left(\left|V\left(C\right)\right|-1\right)},\;\text{if}\;\left|V\left(C\right)\right|>1. \end{array}$$

The density of the subgraph *C* could be looked as the intracluster similarity. Good clustering should have high intracluster similarity and low intercluster similarity. If all nodes in *C* belong to the same cluster, then density(*C*) should be large.

As discussed in Section 2, the centrality of a vertex measures the relative importance of the vertex within the network. One would expect that after clustering, each group has a center and the center has relative high centrality score. On the other side, if a clustering algorithm starts from the vertex (called it a “LEADER”) with high centrality score, one would expect those vertices with tight connection with the LEADER to be grouped together. The clustering result will have high intrasimilarity and low intersimilarity. This is the motivation of the CGC algorithm. Denote the centrality score of the vertex *v* in the graph *G* as score(*v*). For any set *S*, denote the number of elements in *S* as |*S*|.

For any vertex *v* ∉ *V*(*C*), the contribution of *v* to *C* is defined as

(6) $$\begin{array}{c} \text{contribution}\left(v,C\right)=\frac{\sum_{u\in V(C)}w\left(uv\right)\;}{\left|V\left(C\right)\right|}. \end{array}$$

A vertex *v* ∉ *V*(*C*) is called a neighbor of *C* if there is a vertex *u* ∈ *C* such that *uv* ∈ *E*(*G*). The vertex *v* is called a *candidate neighbor* of *C* if *v* satisfies the following three conditions:

(a) *v*  is a neighbor of the subgraph *C*;
(b) there exists a vertex *u* ∈ *V*(*C*), such that
  (7) $$\begin{array}{c} w\left(u,v\right)\geq \alpha *\max\left\{w\left(e\right)\;|\;e\in E\left(G\right)\right\},\;\text{if}\;\left|V\left(C\right)\right|=1,\\ \text{contribution}\left(v,C\right)>\beta *\text{density}\left(C\right),\;\text{if}\;\left|V\left(C\right)\right|>1; \end{array}$$
(c) score(*v*) < max⁡{score(*u*) | *u* ∈ *C*}.

The set of all candidate neighbors of the subgraph *C* is denoted as *N*(*C*).

Condition (a) says that a vertex must be a neighbor of the subgraph *C* in order to be considered to be clustered into the current group *C*. Condition (b) is to control the density of the subgraph *C* such that the density will not decrease too much after the candidate neighbor is added into the subgraph *C*. Condition (c) says that only those vertices with centrality score lower than the centrality score of *some* vertex in *C* are considered. That is, if a vertex *v* ∈ *N*(*C*) has higher centrality score than any vertices in *C*, then the vertex *v* must have already been clustered into another group, so *v* will not be grouped into the group *C*. *α* and *β* are used to control the clustering so that the density of the new subgraph will not decrease too much after a *candidate neighbor* is added into the subgraph *C*. In another paper [18], we proved that if *α* = 0.8 and *β* = 1 − (1/(2∗(|*V*(*C*)|+1))), then the density of the new subgraph with a set of candidate neighbors added to the subgraph *C* will not decrease over 1/3.

The overall structure of the CGC algorithm is shown in Algorithm 1. The three main steps are GROUPING, MERGING, and CONTRACTION.

The details of the GROUPING step is shown in Algorithm 2. The GROUPING algorithm creates a new group from the vertex with the highest centrality score which has not been clustered into any group yet. And this vertex is called the center (or leader) of the new group. Denote this vertex as the center of current group *C*_*i*_. Then the next vertex is chosen from the candidate neighbor set *N*(*C*_*i*_) with the largest contribution to *C*_*i*_.

In the CGC algorithm, every vertex is allowed to be belonged to more than one group. So after the GROUPING step, different groups may have some overlapping elements. If the number of overlapping elements in two groups exceeds some threshold, it is better to merge all vertices in the two groups into a new larger group. The following criterion is applied to determine whether two groups should be merged. Given any two groups, say *C*_*i*_ and *C*_*j*_, if *C*_*i*_ and *C*_*j*_ satisfy the following criterion, then *C*_*i*_ and *C*_*j*_ are merged into one group:

(8) $$\begin{array}{c} \frac{\left|C_{i}\cap C_{j}\right|}{\min\left\{\left|C_{i}\right|,\left|C_{j}\right|\right\}}\geq \frac{1}{2}. \end{array}$$

That is, if the size of overlapping of two groups is greater than half of the size of the smaller one of the two groups, the two groups are merged into one group. The MERGING algorithm (see Algorithm 3) describes the details about how to merge two groups.

After the MERGING step, each group *C*_*i*_ is contracted into a new vertex *v*_*i*_. If there is an edge between two groups *C*_*i*_ and *C*_*j*_, then there will be an edge *v*_*i*_ *v*_*j*_ in the contracted graph. The weight of the edge, *w*(*v*_*i*_, *v*_*j*_), is calculated as follows:

(9) $$\begin{array}{c} w\left(v_{i},v_{j}\right)=\frac{\sum_{e\in E(C_{i},C_{j})}w\left(e\right)\;}{\left|V\left(C_{i}\right)\right|*\left|V\left(C_{j}\right)\right|}, \end{array}$$

where *E*(*C*_*i*_, *C*_*j*_) is the set of crossing edges, *E*(*C*_*i*_, *C*_*j*_) = {*xy* : *x* ∈ *V*(*C*_*i*_), *y* ∈ *V*(*C*_*j*_), *x* ≠ *y*}. The details are presented in the CONTRACTION algorithm (see Algorithm 4).

## 4. Results and Discussion

To evaluate the effectiveness of the CGC algorithm, three benchmark datasets on social network analysis are tested. The three benchmark datasets and the clustering results are described in Sections 4.1, 4.2, and 4.3. The betweenness centrality is used to calculate centrality scores in the CGC algorithm. The results of the CGC algorithm are compared with the ground truth and the results of the Girvan-Newman algorithm [10]. The Girvan-Newman algorithm is one of the most popular algorithms for detecting communities in complex systems. The communities are detected by calculating the edge betweenness centralities of all edges and removing the edge with the highest betweenness value recursively.

To test whether the centrality measures will influence the results, different centrality measures are applied to the CGC algorithm independently and the clustering results are compared in Section 4.4. All of the three datasets could be downloaded from Newman's website [19].

### 4.1. Zachary's Karate Club

**Figure 1:**
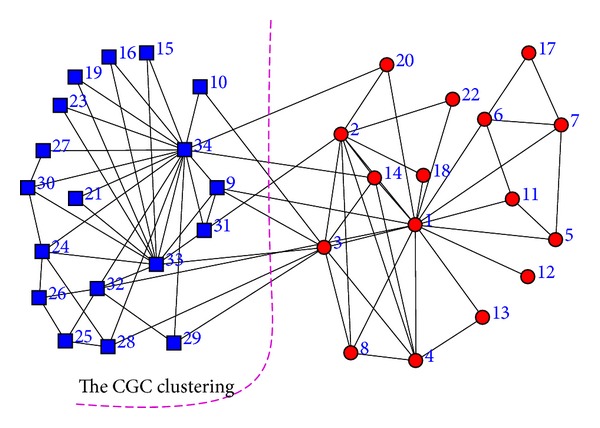
The social network of Zachary's karate club. Red dots denote the supporters of instructor and blue squares denote the supporters of the president. The dashed curve is the partition by the CGC algorithm.

Zachary's karate club dataset is a typical dataset which is used to test the clustering algorithm in social network analysis. It is a social network of friendships between 34 members of a karate club at a US university [20]. Zachary recorded the interaction of the karate club in the university for three years. The social network of relationships in Zachary's karate club is shown in Figure 1. Node 1 represents the instructor of the club and node 34 represents the president of the club. During the observation, there was an incipient conflict between the instructor and the president. And the conflict subsequently led to a formal separation of the club into two organizations: one group is the supporters of the instructor and the other group is the supporters of the president. The ground truth groups are denoted as red dots and blue squares in Figure 1. The red dots denote the supporters of instructor and the blue squares denote the supporters of the president.

**Figure 2:**
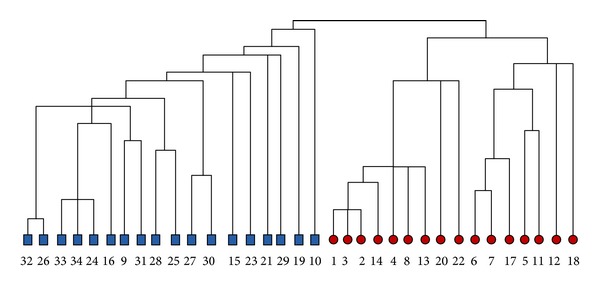
The dendrogram of the karate club dataset by the CGC algorithm.

 When the Girvan-Newman algorithm is applied to this dataset, node 3 is misclassified. The partition by the CGC algorithm is shown as the dashed curve in Figure 1, which is exactly the same as the ground truth.  Figure 2 is the dendrogram corresponding to the result of the CGC algorithm. Another important observation is that when the betweenness centrality is used, the node with the highest betweenness centrality scores is node 1 and the second highest is node 34, which are the instructor and the president, the true leaders of the two groups.

### 4.2. Dolphin Social Network

**Figure 3:**
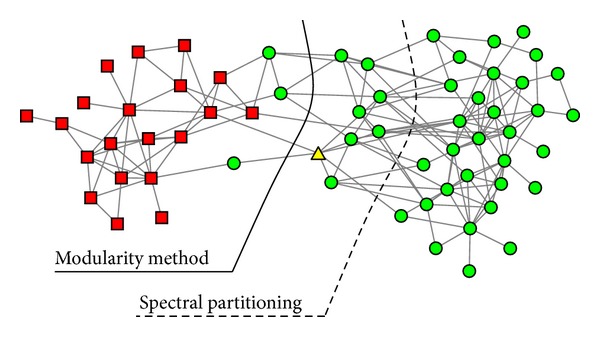
The social network of the dolphins. The dashed curve denotes the division of the network into two equal-size groups found by the standard spectral partitioning method, and the solid curve represents the division found by the modularity-based method by Newman [11].

The dolphin social network dataset is another representative dataset to test the accuracy of clustering algorithms. It is a social network of frequent associations between dolphins in a community in Doubtful Sound, New Zealand [21]. The social network of the dolphins is presented in Figure 3. There are 62 vertices and 159 edges in the network. The vertices represent the bottlenose dolphins, and the edges between the vertices represent associations between dolphin pairs occurring more often than expected by chance. During the course of the study, the dolphins split into two groups following the departure of a key member (represented as the yellow triangle in the Figure 3) of the population.

**Figure 4:**
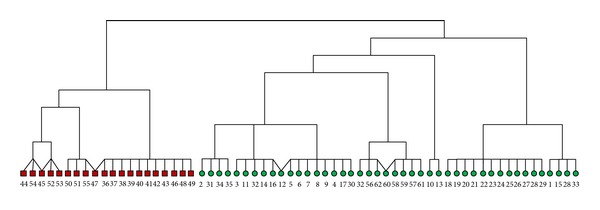
The dendrogram of the dolphin dataset by the CGC algorithm.

The ground truth groups are represented by the shapes of the vertices in Figure 3. The vertices represented as squares are in one group and the vertices represented as dots and triangle are in the other group. The dashed curve represents the division of the network into two equal-size groups found by the standard spectral partitioning method proposed by Newman [11]; 11 out of 62 dolphins are misclassified. The solid curve represents the division found by the modularity-based method by Newman [11]; 3 out of 62 dolphins are misclassified. When the Girvan-Newman algorithm is applied to this dataset, 2 out of 62 dolphins are misclassified. When the CGC algorithm is applied to the dolphin social network, it divides the dolphins into two groups, which is exactly the same as the ground truth. The corresponding dendrogram produced by the CGC algorithm is shown in Figure 4.

### 4.3. Social Network of Political Books

**Figure 5:**
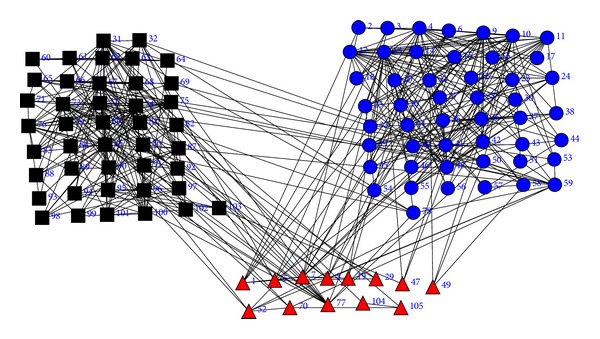
The ground truth partition of the political books. Triangles for neutral books, dots for conservative books, and squares for liberal books.

The third example is a social network map of political books based on purchase patterns from the online book seller Amazon.com. This dataset is provided by Krebs [22]. And the groups of different books are shown in Figure 5. The 105 nodes represent 105 books about US politics. Each book is manually labeled as liberal, neutral, or conservative. Correspondingly, the three types of books are illustrated using three different shapes: triangles for neutral books, dots for conservative books, and squares for liberal books, as in Figure 5. For simplicity, the 105 books are denoted as 1 to 105 instead of book names. Two books are linked in the social network if they were frequently copurchased by the same customer. Figure 5 shows the ground truth classification for the 105 books.

**Figure 6:**
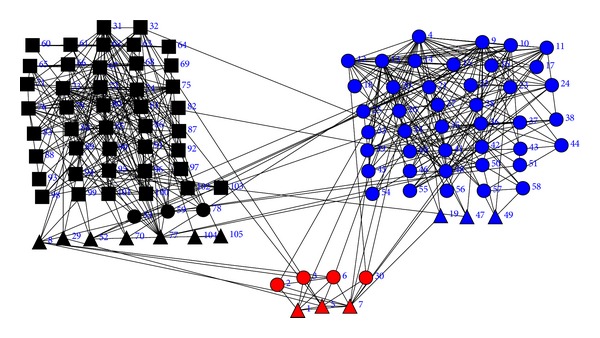
The clustering result of the political books by the Girvan-Newman algorithm. Red for neutral books, blue for conservative books, and black for liberal books.

**Figure 7:**
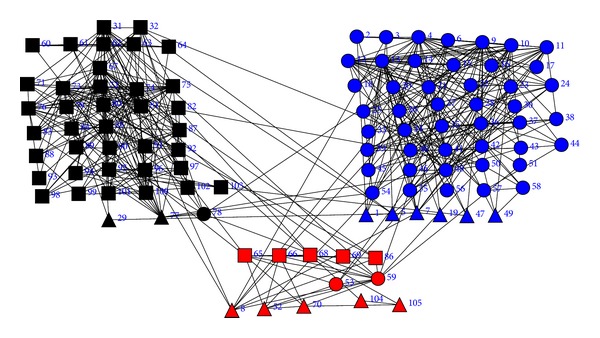
The clustering result of the political books by the CGC algorithm. Red for neutral books, blue for conservative books, and black for liberal books.

In order to see the clustering results based on the book copurchase information, the Girvan-Newman algorithm [10] and the CGC algorithm are applied independently to the adjacency matrix of the political books. When the Girvan-Newman algorithm is applied to the adjacency matrix of the social network, 17 books (2, 3, 6, 8, 19, 29, 30, 47, 49, 52, 53, 59, 70, 77, 78, 104, and 105) are misclassified. The clustering result of the Girvan-Newman algorithm is shown in Figure 6. When betweenness centrality is used and the CGC algorithm is applied to the same dataset, only 16 books (1, 5, 7, 19, 29, 47, 49, 53, 59, 65, 66, 68, 69, 77, 78, and 86) are misclassified. The clustering result of the CGC algorithm is shown in Figure 7.

### 4.4. Clustering with Different Centrality Measures

As mentioned in previous sections, the centrality score of a node in a network could be looked as how important a node is in the network. And the importance of the nodes could be sorted by their centrality scores from large to small. When different centrality measures are applied to the same dataset, the ordering of nodes may be different.

The purpose of this subsection is to test whether the starting clustering node will influence the final clustering result and to compare the effectiveness of different centrality measure when combined with the CGC algorithm. In this subsection, degree centrality, eigenvalue centrality, and betweenness centrality are independently applied to the CGC algorithm. And the same three datasets as in Sections 4.1, 4.2, and 4.3 are used in the experiments.

Table 1 lists the number of misclassified nodes when different centrality measurements are applied to the CGC algorithm. From the table, one could observe that the initial starting node do influence the final results. For the Zachary's karate club dataset, the three centrality measures all produce perfect results. The degree centrality works better than eigenvalue centrality on the dolphin dataset. But on the political book dataset, the degree centrality is worse than the eigenvalue centrality. Overall, the betweenness centrality measure works best with the CGC algorithm.

## 5. Conclusions

In this work, the importance of the centrality score of vertices in a network is discussed and a centrality guided clustering method is proposed. The CGC algorithm initiates the clustering process at a vertex with highest centrality score, which is a potential leader of a community. The CGC algorithm is applied to several benchmark social network datasets. Experimental results show that CGC algorithm works well on social network clustering.

Centrality measurements may influence the results of the CGC algorithm. The degree criterion serves as a very local measurement for centrality, while betweenness centrality and eigenvalue centrality search for global “leaders” of the entire networks. The experiment results show that the betweenness centrality works better than the other two centrality measures for the CGC algorithm.

One may notice that in Figure 4, one single node, such as nodes 45, 47, 12, and 60 in the lowest level, is clustered into two different groups. In fact, it is reasonable for some individual to belong to two different groups. Say for example, some people may be affiliated with two or more organizations. In fact, allowing one object to be clustered into two or more groups is one of the properties of the CGC algorithm, which makes the CGC algorithm different from other clustering algorithms.

The CGC algorithm is a hierarchical clustering algorithm. One direction for future research would be to apply the centrality score guided idea to other clustering methods such as *K*-means clustering. Hopefully, it will also produce promising clustering results.

## Figures and Tables

**Algorithm 1:**
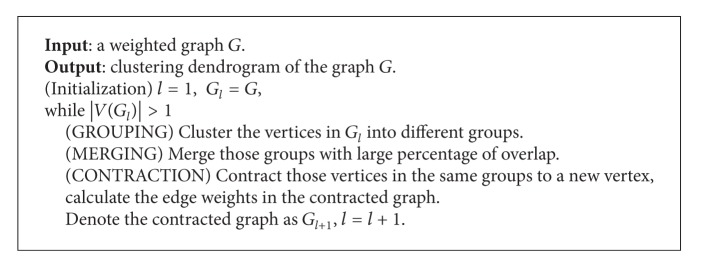
CGC algorithm.

**Algorithm 2:**
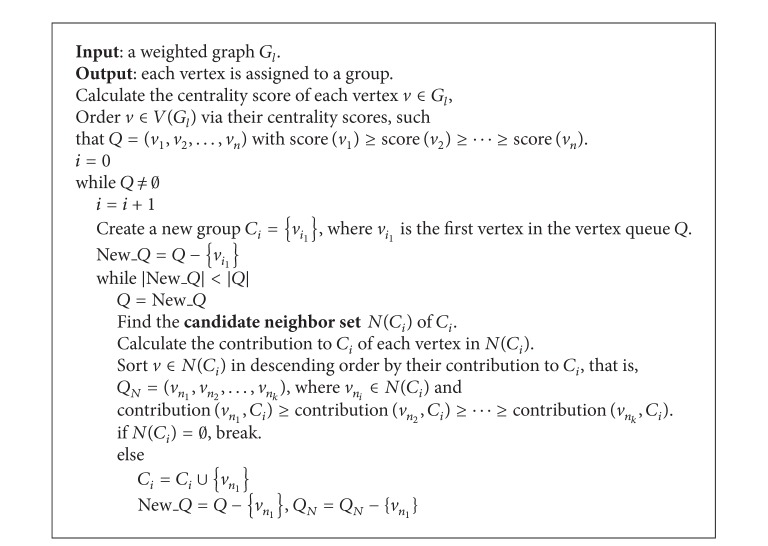
GROUPING algorithm.

**Algorithm 3:**
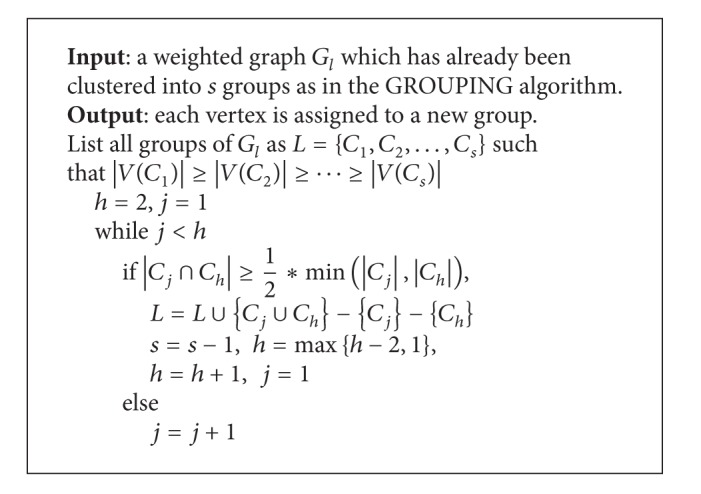
MERGING algorithm.

**Algorithm 4:**
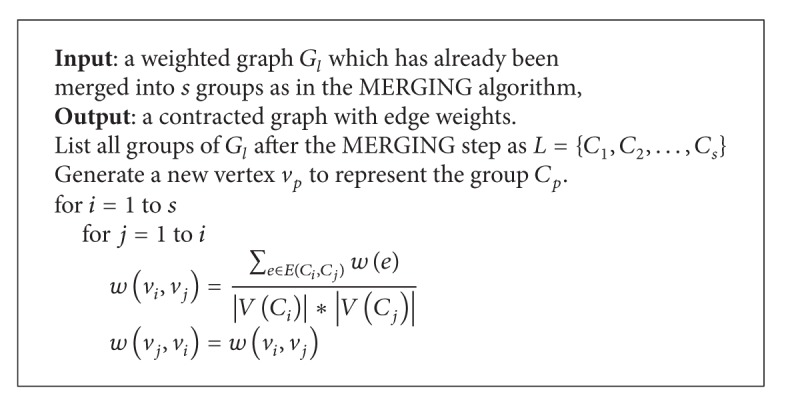
CONTRACTION algorithm.

**Table 1:** The number of misclassified members by the CGC algorithm based on different centrality measures.

	Karate club	Dolphin	Political books
Degree	0	1	17
Eigenvalue	0	2	16
Betweenness	0	0	16
